# Bioprospecting Heavy‐Metal Rhizospheres for Novel Therapeutics Against High‐Priority Multi‐Drug‐Resistant *Pseudomonas aeruginosa* and *Acinetobacter baumannii* : A Case of Toxic to Treatment

**DOI:** 10.1111/1758-2229.70182

**Published:** 2025-09-18

**Authors:** Kylah B. Millard, John O. Unuofin, Luke Invernizzi, Michael O. Daramola, Samuel A. Iwarere

**Affiliations:** ^1^ Sustainable Energy and Environment Research Group (SEERG), Department of Chemical Engineering, Faculty of Engineering, Built Environment and Information Technology (EBIT) University of Pretoria Pretoria South Africa; ^2^ Biodiscovery Centre, Department of Chemistry, Faculty of Natural and Agricultural Sciences (NAS) University of Pretoria Pretoria South Africa

**Keywords:** *Acinetobacter baumannii*, antibiotic resistance, antimicrobial, environmental stress, *Pseudomonas aeruginosa*, rhizospheric microorganisms

## Abstract

This study investigated the antimicrobial potential of rhizospheric microbiota isolated from heavy‐metal‐contaminated soils against two extremely drug‐resistant (XDR) pathogens, *Pseudomonas aeruginosa* (strain ATCC 27853) and *Acinetobacter baumannii* (strain ATCC‐BAA‐1605). Heavy‐metal‐contaminated rhizospheres were sequentially exposed to ex‐situ and in vitro enrichment with heavy metals from battery waste and incubated for 168 h. The surviving microbiota were screened against *P. aeruginosa* and *A. baumannii* , and crude extracts of high‐performing strains were tested against the pathogens using agar well diffusion assays. The novelty and components of the extracted secondary metabolites from environmentally stressed rhizospheric microorganisms were inferred using ultra‐performance liquid chromatography‐high resolution mass spectrometry (UPLC‐HRMS). Results indicated that these secretions inhibited the growth of XDR pathogens (approximately 3.0 × 10^8^ CFU/mL), forming inhibition zones of up to 50 mm. Furthermore, the pathogens were more responsive to exudates from microbiota in environmentally stressed rhizospheres compared to those from organic rhizospheres (control). Heavy‐metal‐stressed microbiota secrete metabolites that show superior antimicrobial activity and successfully inhibit the growth of XDR pathogens. The UPLC‐HRMS analysis indicated the tentative characterisation of the metabolites, particularly *Tolyposamine* and *Gentiatibetine*, by the evaluated microbiota, suggesting their relevance as biopharmaceuticals, and could lead to future antibiotic production.

## Graphical abstract

Heavy metal stress induced the secretion of metabolites not secreted by non‐stressed microbiota. Heavy metal‐stressed microbiota secrete metabolites that inhibit XDR pathogens effectively. UPLC LC–MS tentatively identified novel metabolites, Tolyposamine and Gentiatibetine. Rhizospheric microbes from contaminated environments hold potential for novel antibiotics.

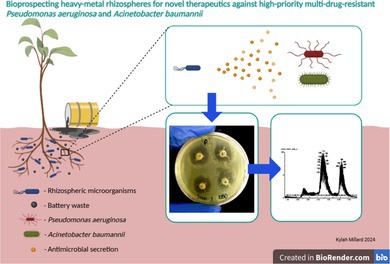

## 1 Introduction

Antibiotic resistance, a phenomenon whereby pathogenic bacteria evolve mechanisms to withstand the bacteriostatic or bactericidal effects of antibiotics, has escalated into a severe public health crisis (Kumar Mehata et al. 2022). This is caused by the widespread and often inappropriate use of antibiotics in human medicine, veterinary practices, and agriculture. Collectively, antibiotic resistance is estimated to cause 700,000 deaths worldwide (Church and McKillip 2021), increase healthcare costs due to longer hospital admissions, higher costs of treatment and unplanned failures, all of which present an economic burden to an already overstretched public health system, especially within the low‐resource setting of Sub‐Saharan Africa (Majumder et al. 2020; Burger and Christian 2020). Addressing antibiotic resistance is imperative, with predictions estimating 10 million deaths globally and a financial cost of 100 trillion USD by 2050 (Kumar Mehata et al. 2022; WHO 2024).

The rise of multi‐drug‐resistant (MDR) and extremely drug‐resistant (XDR) pathogens poses the most pressing threat, as they are often very difficult or impossible to treat with historical therapeutics. Among the most prevalent extremely drug‐resistant (XDR) pathogens are *Pseudomonas aeruginosa* (strain ATCC 27853) (Mini et al. 2022; Kunz Coyne et al. 2022) and *Acinetobacter baumannii* (strain ATCC‐BAA‐1605) (WHO 2024; Mirzaei et al. 2020). The multi‐drug‐resistant strain of *P. aeruginosa* is highly resistant to carbapenem antibiotics, including imipenem and meropenem, placing it among the high‐priority pathogens for antimicrobial resistance intervention (WHO 2024). This resistance complicates treatment, especially in intensive care units, where *P. aeruginosa* is prevalent among immunocompromised patients (WHO 2024). The strain's resistance extends to other beta‐lactams, further reducing effective therapeutic options (WHO 2024). Although carbapenem resistance limits frontline antibiotics, certain combinations like ceftazidime‐avibactam have demonstrated partial efficacy against multidrug‐resistant strains (WHO 2024). Fluoroquinolone resistance, particularly to ciprofloxacin, is also notable in this strain, presenting additional treatment challenges (WHO 2024). Polymyxins such as colistin are sometimes used as alternative agents, although their toxicity restricts their application (WHO 2024).

Likewise, *A. baumannii* exhibits extensive resistance to carbapenems, including imipenem and meropenem, which complicates treatment options, especially in intensive care settings. This resistance is largely due to carbapenemases, making the strain a critical priority for new antimicrobial development (WHO 2024). Additionally, ATCC‐BAA‐1605 shows resistance to aminoglycosides and fluoroquinolones, further limiting effective treatments (WHO 2024). Polymyxin antibiotics like colistin are sometimes effective, though they come with significant toxicity issues (WHO 2024). Combination therapies, such as colistin with tigecycline, have shown variable success in managing infections from this strain (WHO 2024). The World Health Organisation categorises carbapenem‐resistant *A. baumannii* (CRAB) as a critical pathogen due to its high mortality rates (especially in South‐East Asia, East Asia and Oceania) and the global healthcare burden it imposes, highlighting the urgent need for stringent infection control and new antibiotic research and development (WHO 2024). Both pathogens form a part of the notorious ‘ESKAPE’ pathogens (*Enterobacterales*, *Staphylococcus aureus* , *Klebsiella pneumoniae* , *Acinetobacter baumannii* , *Pseudomonas aeruginosa* and *Enterobacter*), the highest‐priority resistant pathogen list as defined by the World Health Organisation (WHO 2024). Such challenges are a threat to human health as a whole.

As much as antibiotic resistance is a threat to human health, heavy metal contamination of the soil has also become a problem as human anthropogenic activity increases (Okereafor et al. 2020). Heavy metals and metalloids with biological toxicity, such as cadmium, iron, arsenic, lead and chromium (Li et al. 2019), pose a serious environmental health risk (Xiang et al. 2021). These heavy metals pose a threat to humans when they are bio‐accumulated in food crops and can result in neurotoxicity, carcinogenesis, cell damage or cellular function loss (Briffa et al. 2020). Despite their toxicity, certain plants have adapted to grow in heavy‐metal‐contaminated soils (Zhou et al. 2021). Moreover, certain plants and their associated microbial communities have adapted to survive in metal‐laden soils containing heavy metals, where the microbes play a key role in supporting plant survival through complex interactions and competitive mechanisms (Hoostal et al. 2008; Khan et al. 2021). The rhizosphere, the narrow zone of soil surrounding the plant roots, is home to complex plant‐microbe‐metal interactions when the plant is under abiotic stress, where environmental stress has been shown to increase the resilience of rhizospheric microbes and promote secondary metabolite production (Hoostal et al. 2008; Yang et al. 2018). In heavy‐metal‐contaminated environments, microbial communities are subjected to extreme selective pressures, which can trigger stress responses in the rhizospheric bacteria (Yang et al. 2018). These stress responses can enhance the distribution of biosynthetic gene clusters (BGCs) responsible for secondary metabolite production, a mechanism through which heavy metal stress could stimulate secondary metabolite production (Tyc et al. 2017). The specific mechanism through which environmental stress impacts the antimicrobial properties of secondary metabolites is unclear. The rhizospheric microorganisms use multiple mechanisms to improve plant tolerance to abiotic stresses, especially heavy‐metal contamination (Khan et al. 2021). These microbes not only facilitate plant tolerance to metal toxicity but also protect the plant through competitive microbial interactions for niches that include secretions of volatile organic compounds (VOCs), siderophores, exopolysaccharides, enzymes and antimicrobials (secondary metabolites) that inhibit growth or encumber survival of pathogenic strains as well as regulate nutrient and heavy metal bioavailability to plants (Zhou et al. 2021; Tyc et al. 2017; Khasheii et al. 2021; Nakayasu et al. 2023).

Given that environmental stress encourages microbial inter‐species competition, and hence resilience and robustness from surviving species, it is unsurprising that extreme habitats have been noted as ‘hot spots’ of novel and conventional microorganisms for investigation through drug discovery programmes (Sayed et al. 2020). The adaptive mechanism of secreting secondary metabolites has been observed in various extreme habitats, such as geothermal springs, deep‐sea hydrothermal vents, desert microbiomes and polar regions, where microbes have evolved to survive under severe ecological stressors (Wen et al. 2022; Baranova et al. 2023). These ecological stressors parallel the selective pressures observed in heavy metal‐contaminated rhizospheres, advocating for the hypothesis that environmental stressors can be leveraged to enhance antimicrobial secretion in microbial communities. Natural extreme habitats have been successfully investigated for novel antimicrobial secretions, including deserts, highly saline salt pans, permafrost soils and deep‐sea sediments (Sayed et al. 2020). A relevant example of this is the secondary metabolites from a *Bacillus* sp., isolated from a salt pan environment, that exhibited moderate antimicrobial activity against pathogens such as *Staphylococcus aureus* and *Escherichia coli*, along with promising antioxidant properties (Sujitha et al. 2024). While no activity was observed against *P. aeruginosa* , the findings highlight the chemical diversity of extremophile‐derived metabolites and their relevance in addressing multidrug resistance. Such studies underscore the rationale for exploring microorganisms from stressed environments as potential sources of novel antimicrobial agents. This approach aligns with the hypothesis that microbes adapted to extreme conditions produce metabolites with enhanced bioactive properties due to competitive survival mechanisms (Sujitha et al. 2024). Along with naturally occurring extreme environments, synthetically stressed environments have also been explored and investigated, including carwash effluent tanks and drains (Sibanda et al. 2017). Investigation of these synthetic environments resulted in multiple compounds being identified with antibacterial, antifungal or anticancer properties (Sibanda et al. 2017). It has been demonstrated in literature that natural products have vast chemical diversity and a range of biological activity, often consisting of complex molecular structures that are often difficult to replicate synthetically, making them a valuable source of new drugs over the past four decades (Newman and Cragg 2020; Genilloud 2019; Butler et al. 2014; Lewis et al. 2024). Fluoroquinolones, tetracyclines and penicillin are among the most well‐known drugs that were successfully developed from natural products (Lewis et al. 2024). Many *Bacillus* strains found in soil have exhibited antimicrobial activity and present an interesting arsenal for novel antimicrobial discovery (Zhou et al. 2021; Todorova and Kozhuharova 2009). In particular, compounds secreted by rhizospheric bacteria are often viewed as attractive options as antimicrobials due to their natural origin, specificity, and diverse modes of action, which enable them to target pathogens while minimising harm to non‐target organisms and the environment (Lewis et al. 2024).

Recent research has also demonstrated the immense potential of industrial wastewater as a reservoir of biologically active secondary metabolites with significant antimicrobial properties (Kumar Mehata et al. 2022). Kumar et al. isolated four bacterial strains—including *Bacillus subtilis*—from industrial effluents, identifying key secondary metabolites such as Carbenicillin, Cephalexin, Cephalothin and Tetracycline through chromatographic and HPLC techniques. These compounds exhibited robust antimicrobial activity against multidrug‐resistant pathogens, including *Shigella dysenteriae* , *Proteus vulgaris* , *P. aeruginosa* , and *Enterococcus faecalis* (Kumar Mehata et al. 2022). Literature highlights anthropogenic environments as a source of bioactive compounds and underscores their relevance in addressing the growing challenge of antibiotic resistance, offering novel agents for targeted therapeutic applications.

As far as it could be ascertained, rhizospheric microorganisms from heavy‐metal‐contaminated soils have not yet been investigated for their ability to secrete secondary metabolites capable of inhibiting the growth of multi‐drug‐resistant pathogens like *P. aeruginosa* (strain ATCC 27853) and *A. baumannii* (strain ATCC‐BAA‐1605). It is hypothesised that microorganisms thriving in these extreme environments, which require resilience and competitive survival mechanisms, produce antimicrobial secretions that may effectively inhibit the growth of multidrug‐resistant human pathogens. Species of *Pseudomonas* are known to exist within plant rhizospheres and soil, so it is plausible that the heavy‐metal‐contaminated rhizospheric bacteria could have evolved the relevant competitive antimicrobials to inhibit these pathogens. Therefore, this study aimed to elucidate the inhibitory capabilities of possible antimicrobial secretions from a quantified heavy‐metal‐contaminated environment against *P. aeruginosa* and *A. baumannii* and to characterise the bioactive molecules tentatively.

## 2 Methodology

### 2.1 Sampling and Conditioning—Description of Location

Experimental rhizospheric soil samples were collected from eight individual plants growing in two distinct heavy metal contaminated environments from a scrapyard in Pretoria, South Africa (Salvage and Recycling Silverton (218 Moreleta St, Koedoespoort 456‐Jr, Pretoria, 0184, 25°43′39.6″ S 28°16′39.6″ E)), with permission granted by the owner.

Six plants were sampled from a tailings site, and two were sampled from a steel dump site. These plants were the only individuals visibly growing in their respective areas, presumably due to the elevated concentrations of heavy metals in the surrounding soil, which limited broader vegetation. These sites were selected based on their history of heavy metal exposure, and the sampling strategy was designed to explore the microbial communities and their potential antimicrobial properties under heavy metal stress, rather than focusing on plant species–specific interactions. The study was therefore conducted in an exploratory framework aimed at environmental bioprospecting for antimicrobial activity.

All eight plant rhizospheres were sampled and treated independently at first, but rhizospheric soils from the same site were later combined into two composite inocula, one for the tailings site and one for the steel dump site. This was done during the selective enrichment phase to focus the study on the effects of environmental stress, not plant species. Pooling facilitated a more robust representation of the environmental microbiome from the contaminated site, improving the diversity of bacteria available for downstream antimicrobial screening.

A control rhizosphere was obtained from a plant (*Lantana* sp. Figure S3) grown in soil free of heavy metals in the gardens of the University of Pretoria. The control plant was identified at the H.G.W.J. Schweickerdt Plant Herbarium at the University of Pretoria and was assigned the voucher specimen number PRU0132960.

The plant specimen that provided the contaminated rhizosphere from which the best‐performing microorganisms were sourced was identified at the H.G.W.J. Schweickerdt Plant Herbarium at the University of Pretoria and was assigned the voucher specimen number PRU0132903, with the name *Erigeron bonariensis* , Figures S4 and S5. The other plant species have yet to be formally identified.

Harvesting of the plants was done by gently uprooting the plants, keeping as much of the rhizospheric soil intact as possible. The plants were then placed into containers and filled with the soil from which they originated. About 10 mL of deionised water was added to keep the plants alive. The plants were left for approximately 168 h to incubate at room temperature under natural light for observation to ensure their stability for future experimentation. The plants were sprinkled with 10 mL of water every second day.

The experimental plants' rhizospheres were then contaminated with battery waste (extracted from a used AA Energiser battery in powder form) at a 0.01% solution of 5 mL per sample from the contaminated soil. A 5 cm diameter around each plant stem was contaminated to establish further competitive exclusion between microbes. The organic sample remained uncontaminated to function as a control. The plants were incubated for 168 h at room temperature under natural light. Afterwards, each rhizospheric sample was collected by carefully excavating soil within a 2 mm radius around the root zone of each plant using a spatula to obtain 1 g of rhizospheric soil for X‐ray fluorescence (XRF) analysis using the Bruker M4 Tornado system. The remaining rhizospheric soil was used for selective microbial enrichment and further downstream bioactivity screening.

### 2.2 Selective Enrichment and Serial Dilution of Rhizospheric Bacteria

Rhizospheric samples were taken using a spatula by scraping the roots of the plants harvested to collect the surrounding soil. Rhizospheric soils from plants sampled at the same location were pooled prior to selective enrichment in order to reflect the collective microbial community associated with each contaminated environment. Specifically, all rhizosphere samples collected from the steel dump site were combined to form a steel‐associated inoculum, and all rhizosphere samples from the tailings site were combined to form a tailings‐associated inoculum. Similarly, the two rhizosphere samples from organically grown plants were pooled to represent the uncontaminated control group. The samples were named ‘Steel’, ‘Tailings’ and ‘Organic’, respectively. This approach was adopted to focus on the influence of environmental heavy metal stress on rhizospheric microbial composition and activity, rather than on plant species–specific interactions. The pooled samples were then subjected to selective enrichment to promote the growth of bacteria adapted to each respective soil environment. Fifteen grams of each rhizospheric soil sample containing rhizospheric bacteria was then placed in a separate flask of selective enrichment media comprising (g/L): MgSO_4_·7H_2_O (purchased from Saarchem, Merck, Lethabong, South Africa, ≥ 95%); 0.50, Na_2_HPO_4_ (purchased from Merck, Lethabong, South Africa, ≥ 95%); 0.12, Na_2_HPO_4_·H_2_O (purchased from univAR, Merck, Lethabong, South Africa, ≥ 95%); 0.20, NaCl (purchased from Glassworld, Johannesburg, South Africa, ≥ 98%); 1.0, KNO_3_ (purchased from Glassworld, Johannesberg, South Africa, ≥ 95%); 1.80, battery waste (from AA Energiser battery); 0.10. The enrichment medium of the control did not contain any battery waste. Each sample was placed in 200 mL of selective enrichment medium and incubated at 28°C, 120 rpm for 144 h. One millilitre (1 mL) of the respective fractions was subsequently serially diluted to 10^−3^, 10^–5^ and 10^−7^ in a sterile 0.76% NaCl solution. Thereafter, 200 μL aliquots were plated onto selective agar comprising (g/L): MgSO_4_·7H_2_O (purchased from Saarchem, Merck, Lethabong, South Africa, ≥ 95%); 0.50, Na_2_HPO_4_ (purchased from univAR, Merck, Lethabong, South Africa, ≥ 95%); 0.12, Na_2_HPO_4_·H_2_O (purchased from univAR, Merck, Lethabong, South Africa, ≥ 95%); 0.20, NaCl (purchased from Glassworld, Johannesberg, South Africa, ≥ 99%); 1.0, KNO_3_ (purchased from Glassworld, Johannesberg, South Africa, ≥ 95%); 1.80, battery waste (from AA Energiser battery); 0.10, tryptone (purchased from LabChem, Johannesberg, South Africa); 0.80, humic acid (purchased from Sigma‐Aldrich Kempton Park, South Africa, ≥ 99%); 0.05, FeSO_4_·7H_2_O (purchased from Sigma‐Aldrich, Kempton Park, South Africa, ≥ 95%); 0.50, C_6_H_12_O_6_ (purchased from Glassworld, Johannesberg, South Africa, ≥ 90%); 2.00, C_4_H_6_MnO_4_ (purchased from Merck, Lethabong, South Africa, ≥ 95%); 0.01, cycloheximide (purchased from BDH Laboratory Supplies, Kampala, Uganda, ≥ 90% by assay); 0.05, agar (Bacteriological from BioLab, Merck, Lethabong, South Africa); 12.0. The plates were incubated for 72 h at 28°C to encourage microbial growth.

Following incubation of the agar plates, distinct bacterial colonies became visible. These colonies were preliminarily differentiated based on observable morphological characteristics, including colony shape, size, colour, elevation, margin and texture. Representative colonies with distinct morphologies were carefully selected to ensure a broad representation of microbial diversity, all from heavy‐metal contaminated soils, thereby increasing the likelihood of capturing strains with unique biosynthetic potential. By maximising phenotypic variation at the early isolation stage, the study aimed to strengthen the diversity of the screened sample set and improve the probability of identifying strains capable of producing bioactive antimicrobial compounds. Each selected colony was then purified through repeated streaking on sterile nutrient agar plates to obtain pure cultures for downstream analysis. Each selected colony was subsequently purified through repeated subculturing using the streak plate method on sterile nutrient agar. This ensured the isolation of single colonies for downstream screening and analysis. The isolates were named according to their environments of origin, where 11 distinct ‘Steel’ isolates were selected, nine distinct ‘Tailings’ isolates were chosen, and 13 distinct ‘Organic’ isolates were selected.

### 2.3 Primary Screening and Fermentation

**FIGURE 1:**
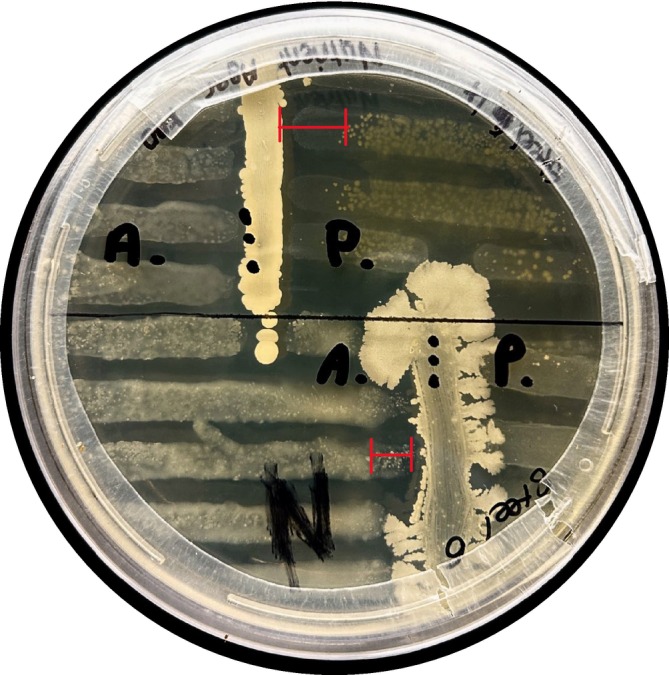
Primary screening of bacterial isolates against *P. aeruginosa* (P) and *A. baumannii* (A) using the perpendicular streak method. Inhibition was defined as a visible reduction or absence of pathogen growth across the interaction zone. Partial inhibition is illustrated by faint residual growth in the *A. baumannii* zone (bottom red bracket), and strong inhibition is demonstrated in the top red bracket. Results shown are from a single representative plate. No replication was performed in this preliminary screen.

Following selective enrichment, bacterial isolates were screened for antimicrobial activity using the perpendicular streak test, where the pure rhizospheric bacteria cultures were streaked vertically against horizontally streaked cultures of *A. baumannii* ATCC BAA 1605 and *P. aeruginosa* ATCC 27853. A single replicate for each was performed. Both pathogens were purchased from Thermo Fisher Scientific in Centurion, South Africa, on a mimic of Mueller–Hinton agar comprising (g/L): nutrient broth (purchased from BioLab, Merck, Lethabong, South Africa); 3.4, casein (purchased from Sigma‐Aldrich, Kempton Park, South Africa); 2.0, and agar (Bacteriological from BioLab, Merck, Lethabong, South Africa); 14.0. The plates were incubated at 28°C for 48 h. Positive screens were identified where it could be seen that the rhizospheric bacteria had created a transparent zone at the site of pathogenic bacterial streaking, as shown by the red lines in Figure 1. Positive screens were further classified as either ‘partial inhibition’ or ‘strong inhibition’, where the distinction was made visually based on reduced turbidity and disrupted colony continuity. Partial inhibition was defined as between 2 and 4 mm inhibition, and strong inhibition was defined as > 4 mm inhibition. If no transparent zones were shown, it was deemed a negative screen. This screening was only performed once as a preliminary, exploratory assay without replication.

Thereafter, isolates that produced positive test results were used to inoculate quarter‐strength nutrient broth (purchased from BioLab, Merck, Lethabong, South Africa) for 120 h at 28°C, 120 rpm, so that they could be further investigated for their antimicrobial‐secreting capabilities.

### 2.4 Secondary Metabolite Extraction

The bioactive compounds were recovered from the cell‐free supernatant (after centrifugation at 8150× g for 9 min) by the method of solvent‐solvent extraction with ethyl acetate (Sigma‐Aldrich, Kempton Park, South Africa, ≥ 95%), acetone (Sigma‐Aldrich, Kempton Park, South Africa, ≥ 95%), dimethyl sulfoxide (Sigma‐Aldrich, Kempton Park, South Africa, ≥ 95%), and acetic acid (Sigma‐Aldrich, Kempton Park, South Africa, ≥ 95%) (1:2:2:1) and allowed to sit in a separating flask for 48 h or until the separate organic layers were visible. Each organic fraction was then extracted into a sterile bottle and subjected to rotary evaporation (Bucci RotaVap R‐300), as adapted from the methods used in literature (Sujitha et al. 2024; Kumar et al. 2021).

### 2.5 Secondary Screening

The extracted organic fractions were tested against 1.0 MacFarland standards of *A. baumannii* ATCC BAA 1605 and *P. aeruginosa* ATCC 27853 purchased from Thermo Fisher Scientific on the Mueller‐Hinton agar (from BioLab, Merck, Lethabong, South Africa). The agar well diffusion test was performed using 500 μL of the concentrated organic fractions as well as the centrifuged fermentation broth, injected in 4 mm diameter wells. The plates were incubated at 25°C for 48 h. A single replicate of each test was performed. Thereafter, the plates were observed for zones of inhibition, where the best performers were selected for further analysis. Control tests were performed with 500 μL of each pure organic solvent to ensure that any activity shown would be as a result of bioactive compounds dissolved within the solvents and not the solvents themselves. The agar diffusion assays were conducted once per isolate as an exploratory screen to assess inhibitory activity; no biological or technical replicates were performed (*n* = 1). The rhizospheric bacteria responsible for producing the secondary metabolites that showed the greatest pathogen inhibition were characterised using 16S rRNA sequencing.

### 2.6 16S rRNA Sequencing

The purified bacterial cultures showing inhibition were streaked onto separate nutrient agar petri dishes using a microbiological hockey stick. The strain identification was based on the ±600 bp partial sequence of the 16S rRNA gene of the organisms. The 16S rRNA gene region was amplified with the universal primers, after which the DNA was extracted, and PCR amplification took place. The sequences were compared against the GenBank of the National Centre for Biotechnology in the United States of America using a basic BLAST search.

### 2.7 Bioactive Screening

Three separate flasks of selective enrichment medium were inoculated with the top three best‐performing isolates—namely Steel 3, Steel 4 and Steel 7, based on activity shown by zones of inhibition—from the previous screening exercise and were incubated for 120 h at 28°C, under agitation of 120 rpm on a shaker bed. Each broth was then centrifuged at 9000 rpm for 12 min, and the supernatants were collected. A rotary evaporator was used to concentrate the samples. After concentration, samples were placed in an evaporating chamber at 38°C to dry the remaining samples. Samples were weighed in powder form. Solubility tests were then performed on the powdered samples using different ratios of water, methanol, and acetonitrile as seen in the Supporting Information, Table S2.

The powders were dissolved in 10% DMSO to obtain a concentration of approximately 5000 μg/μL. *P. aeruginosa* ATCC 27853 was streaked onto the Muller–Hinton agar, and 100 μL of each sample was injected into each well. The petri dishes were sealed with parafilm and left to incubate over 24 h at 36°C. A positive control of 500 μg Gentamicin (Virbac, Centurion, South Africa) was used on the same agar under the same incubation conditions as the experimental samples. A single replicate of each test was performed.

### 2.8 UPLC‐HRMS Analysis

The bioactive powders were dissolved in a MeOH: H_2_O (1:1) (both chemicals obtained from Romil Pure Chemistry, HPLC grade) solution to obtain a concentration of 1000 ppm. To ensure no particulate matter was present, the samples were filtered using a 0.22 μm nylon filter. Only the extract from Steel 7, which displayed the largest and most potent inhibition zones during the primary and secondary screening, was subjected to UPLC–MS analysis. The extracts from Steel 3 and Steel 4 were stored but not analysed further. This selective profiling allowed for the identification of candidate bioactive compounds from the most promising isolate while maintaining the integrity of individual strain comparisons throughout the screening process.

The UPLC analysis was conducted using a Waters Acquity UPLC system (Waters Corp.) featuring Aquity PDA and Xevo G2 qTOF detectors. This system was equipped with both a binary solvent delivery unit and an autosampler. A leucine enkephalin solution at 5 ng/μL served as the lock spray solution, infused continuously at 2 μL/min through a separate orthogonal ESI (Electrospray Ionisation) probe to maintain mass accuracy by compensating for any experimental drift. Leucine enkephalin was purchased from Waters via Microsep (South Africa) as an internal calibrant for the monoisotopic mass analysis.

Sample separation utilised an XBridge UPLC C18 column (2.1 × 150 mm, 1.7 μm) from Waters Inc. (Milford, MA, USA). The mobile phase consisted of solvent A: 0.1% formic acid in HPLC‐grade water and solvent B: acetonitrile containing 0.1% formic acid. The flow rate was set to 0.3 mL/min, with an injection volume of 5 μL, and the column was maintained at 50°C.

A Waters Synapt G2 high‐definition QTOF mass spectrometer, equipped with an ESI source, was utilised to gather data in both negative and positive ion modes. The system operated under MassLynx V 4.1 software (Waters Inc., Milford, Massachusetts, USA).

Monoisotopic mass analysis was carried out in both ESI(+) and ESI(−) ionisation modes, with capillary voltages set to 2.80 and 2.0 kV, respectively. A mass scan range of 50–1200 Da was used, with a full width at half maximum (FWHM) mass resolution of 22,000. The source temperature was maintained at 120°C, while the desolvation gas temperature was set at 350°C. To ensure consistent results despite any potential mass accuracy drift, leucine enkephalin (m/z 555.2693) was intermittently infused at a constant flow rate of 3 μL/min every 10 s.

For UV detection of the eluting analytes, the Aquity PDA was configured with a scan range from 220 to 700 nm, using a scan time of 0.05 s and a full width at half maximum (FWHM) mass resolution of 4.8 nm.

The top two major peaks evident in the LC–MS chromatogram were selected for tentative identification. Tentative identification included determining the accurate mass formula, monoisotopic mass, molecular mass error and the UV activity of the compounds using MassLynx software. Lock mass correction was accounted for in both negative (554.2615) and positive (556.2771) modes. The adducts were accounted for in positive and negative modes. Fragment combinations and fragment identity were confirmed using PubChem, ChemDraw and ChemNetBase. The Lotus natural products database was utilised to check for known natural products produced by the rhizospheric bacteria identified by the 16S rRNA sequencing.

Compounds were tentatively identified by generating molecular formulas from MassLynx V 4.1 based on their iFit value, and by comparison of MS/MS fragmentation patterns with those of matching compounds from PubChem (Kim et al. 2023). Additionally, acquired accurate masses were compared with those of known compounds in compound databases.

## 3 Results and Discussion

### 3.1 Heavy‐Metal Pollution and Bacterial–Metal Interaction

It was observed that metallic elements were prevalent in all the samples evaluated, ranging from critical to non‐critical concentrations (Figure S2). In particular, some notable metal concentrations beyond the national acceptable standard were highlighted in Table 1, further substantiating the incidence of metal contamination, which might stimulate physiological and metabolic responses of both flora and fauna. Models of metals with such activity are cadmium (Cd), arsenic (As), lead (Pb) and chromium (Cr) (Li et al. 2019). Correspondingly, this study reports arsenic and chromium at critical levels well beyond recommended national standards, which are 0.002 ppm As, 0.06 ppm Cr^6+^ and 4.56 ppm Cr^3+^, respectively (Al‐Makishah et al. 2020; Liu et al. 2021). Inorganic forms of arsenic are more phytotoxic, carcinogenic, and bio‐toxic at low concentrations than organic forms, and are mostly found in soils, where they might interchange between As^3+^ and As^5+^ under anaerobic and aerobic conditions, respectively (Rajendran et al. 2024). Although Cr^6+^ is involved with the electroplating of steel and alloys, certain biogeochemical processes might also facilitate the spontaneous, reversible conversion of naturally occurring Cr^3+^ to Cr^6+^ in the soil, especially in the presence of manganese oxide and soil carbon compounds (Ao et al. 2022; Mortada et al. 2023). The contaminated site in the present study has a history of perpetual stockpiling of steel wastes, among other electronic wastes, so it is not unreasonable to assume the incidence of constant interconversions between Cr^3+^ and Cr^6+^. Moreover, it was found in concentrations geometrically higher than national acceptable standards (Table 1). Due to its strong carcinogenicity and teratogenicity, Cr^6+^ species are classified as Class A Human Carcinogens by the United States Environmental Protection Agency (US EPA) and are particularly injurious to plants (Wang et al. 2024). Interestingly, the plants evaluated in this study were able to survive such a polluted environment and also tolerated additional metal stress ex situ, thereby suggesting a plant‐microbe‐metal interaction that enabled their survival. One of such benefits is the biotransformation of toxic metals by rhizospheric microbiota, reducing their bioavailability to the plants. This phenomenon was inferred by an XRD analysis (Figures S1 and S2), which showed a decreased intensity or dramatic disappearance of peaks after 144 h of additional exposure to battery waste. This phenomenon might be due to the precipitation of certain heavy metals inside the cells of the rhizospheric microbiota, which has been corroborated by studies involving individual metals or cocktails of heavy metals (Han et al. 2020; Wang et al. 2022; Gupta et al. 2024; Narenkumar et al. 2024). Moreover, this bacterial‐metal interaction was not only observed to reduce the uptake of Cd and Pb by spinach in vegetable fields contaminated with heavy metals (Wang et al. 2022) but also stimulated the intense antibiosis of an *Actinobacterial* strain against *Escherichia coli* , *Mycobacterium smegmatis* , *Staphylococcus aureus* and *Candida albicans* (Haferburg et al. 2009).

**TABLE 1:** Elemental concentrations of selected toxic heavy metals in steel‐contaminated soil, tailings‐contaminated soil, and organic soil as determined by X‐ray fluorescence (XRF) analysis.

Toxic element	Steel soil (ppm)	Tailings soil (ppm)	Organic soil (ppm)	Acceptable standard (ppm)
Arsenic	24.3	9.2	3.4	0.002 (Al‐Makishah et al. 2020)
Cadmium	1.0	1.6	< 0.1	0.3 (Wu et al. 2021)
Chromium	643.8	658	166.9	0.60 (VI) and 4.51 (III), respectively (Li et al. 2019)
Lead	103.8	76.3	18.5	200 (Kinuthia et al. 2020)

*Note:* Concentrations are expressed in parts per million (ppm). Acceptable soil contamination standards for each element are indicated for reference, with relevant citations.

A more detailed elemental analysis is presented in Table S1 of the Supporting Information, which shows the concentration of each element present in the different soil types.

### 3.2 Isolation and Purification of Rhizospheric Bacteria

A total of 33 distinct bacterial colonies were isolated from environmentally stressed soil (20) and organic, non‐environmentally stressed soil (13), which exhibited distinct colony morphologies. Table 2 reports CFU counts derived from composite rhizosphere samples. Rhizospheres from the steel dump site were pooled to create a representative ‘Steel’ sample, and those from the tailings site were pooled to create a ‘Tailings’ sample. Each composite was serially diluted (10^−3^, 10^−5^ and 10^−7^) and plated in triplicate (*n* = 3) to ensure technical replication. The CFU values presented are based on the 10^−3^ dilution, although data from other dilutions supported the observed trends. CFU counts from the Steel and Tailings composites are shown separately in Table 1 to preserve their individual contributions, and these values were then averaged to generate the overall ‘Contaminated’ group. This approach reflects the study's focus on microbial viability in response to environmental heavy metal stress, rather than plant‐specific microbiomes.

**TABLE 2:** Colony‐forming unit (CFU) counts obtained from composite rhizospheric samples of steel‐contaminated, tailings‐contaminated, and organic soils.

CFU origin	Plate dilution × 10^−3^	Plate dilution × 10^−5^	Plate dilution × 10^−7^	Average combined CFU × 10^4^ mL^−1^
Steel rhizosphere	46	3	0	173
Tailings rhizosphere	72	7	0	386
Organic rhizosphere	103	9	3	10,334

*Note:* CFU counts are shown for three serial dilutions (10^−3^, 10^−5^ and 10^−7^). Average CFU counts (×10^4^ CFU/mL).

It is notable that a substantially higher number of colony‐forming units (CFUs) was recovered from the organic rhizosphere samples (10,334 × 10^4^ CFU/mL) compared to the heavy‐metal‐contaminated rhizospheres (173 × 10^4^ and 386 × 10^4^ CFU/mL for Steel and Tailings, respectively). The marked difference observed suggests that microbial communities in contaminated soils are subjected to significant environmental stress, likely suppressing sensitive taxa and allowing the survival of only the most resilient microorganisms. Heavy metals such as arsenic, cadmium and chromium are known to disrupt essential cellular processes, including enzyme function, membrane integrity and DNA replication, all of which can contribute to cellular toxicity and microbial mortality (Balali‐Mood et al. 2021). The observed reduction in colony numbers may therefore reflect a selective bottleneck imposed by metal toxicity, whereby only stress‐adapted microbes with resistance mechanisms survive.

This observation aligns with the concept of competitive exclusion under stress conditions where microbial diversity is constrained, and ecological niches are dominated by a few specialised organisms. These findings are consistent with prior studies showing that heavy metal contamination reduces total microbial biomass and alters community structure, often leading to diminished ecosystem functionality and resilience (Zhao et al. 2020; Shuaib et al. 2021). The reduced CFU counts observed in this study may also indicate an ecological cost associated with heavy‐metal resistance, as energy‐intensive stress responses can reduce overall microbial growth rates and reproductive success. Together, these results highlight the significant ecological impact of soil contamination and reinforce the importance of targeting stress‐adapted microbial populations for antimicrobial screening efforts.

The combination of the XRF analysis and the reduced CFU count suggests that environmental stress in the form of heavy‐metal contamination is present, and that the surviving strains might have exhibited certain physiological and metabolic traits to consolidate their resilience. Correspondingly, literature indicates that environmental stress increases the resilience of the surviving microbes and that the production of secondary metabolites is often triggered by environmental stress (Hoostal et al. 2008; Tyc et al. 2017).

### 3.3 Primary Screening—Perpendicular Streak Method

Of the 33 distinct isolates tested against both XDR pathogens (Table 1), four isolates showed inhibition against both, whereas six isolates showed inhibition against either of the pathogens, as demonstrated in Figure 1. The bacterial isolates were named ‘Steel’ or ‘Tailings’ if they originated from environmentally stressed rhizospheres (sampled from an old steel dump or an iron tailings dump, respectively), and were named ‘Organic’ if they originated from the control, a non‐environmentally stressed rhizosphere. The numbers were assigned based on the order in which they were removed from the original CFU count plates, showing the results of the serial dilution, and do not indicate anything else.

Table 3 summarises the inhibitory effects of 33 distinct bacterial isolates, derived from steel dump, tailings, and organic rhizospheres, against two high‐priority multidrug‐resistant pathogens: *P. aeruginosa* and *A. baumannii* . A clear trend emerges in which isolates from the steel‐contaminated rhizospheres exhibited the highest levels of antimicrobial activity. Specifically, 7 out of the 11 steel isolates demonstrated inhibition against at least one pathogen, with Steel 7 showing strong inhibition against both pathogens, making it the most promising candidate for downstream investigation.

**TABLE 3:** Results of the Primary screening where Partial inhibition indicates a change in the density and a disruption of continuity of pathogen growth along the streak path adjacent to the test isolate of between 2 and 4 mm, and Strong Inhibition was defined as 4 mm or more.

Distinct colony	*Pseudomonas aeruginosa*	*Acinetobacter baumannii*
Steel 1	Partial inhibition	None
Steel 2	Strong inhibition	Partial inhibition
Steel 3	Partial inhibition	Strong inhibition
Steel 4	Strong inhibition	Partial inhibition
Steel 5	None	None
Steel 6	None	Partial inhibition
Steel 7	Strong inhibition	Strong inhibition
Steel 8	None	None
Steel 9	None	None
Steel 10	None	None
Steel 11	Partial inhibition	None
Tailings 1	None	None
Tailings 2	None	None
Tailings 3	None	None
Tailings 4	None	Partial inhibition
Tailings 5	None	None
Tailings 6	None	None
Tailings 7	Partial inhibition	None
Tailings 8	None	None
Tailings 9	None	None
Organic 1	None	None
Organic 2	None	None
Organic 3	None	None
Organic 4	None	None
Organic 5	Partial inhibition	None
Organic 6	None	Partial inhibition
Organic 7	None	None
Organic 8	None	None
Organic 9	None	None
Organic 10	None	None
Organic 11	None	None
Organic 12	None	None
Organic 13	None	None

Several other steel isolates, including Steel 2, Steel 3 and Steel 4, also displayed combinations of partial and strong inhibition, further supporting the hypothesis that chronic exposure to heavy metal stress selects for metabolically robust and competitive bacterial strains capable of producing bioactive secondary metabolites. This trend is consistent with the concept of competitive exclusion under abiotic stress, wherein only microbial strains with advanced adaptive traits, such as antimicrobial secretion, can survive and dominate. Inter‐species interactions and competition under nutrient‐limited conditions are one of the main biotic factors affecting the production of secondary metabolites and might explain the secretion of antimicrobials against the multidrug‐resistant pathogens (Tyc et al. 2017). Moreover, literature has shown that soil bacteria growing under environmental stress can be specifically triggered to produce broad‐spectrum antibiotics when challenged by other bacterial species, which supports the results observed (Tyc et al. 2017). The steel dump site may thus provide a particularly selective environment due to the higher observed metal concentrations, as discussed in previous sections.

In contrast, isolates from the tailings site demonstrated notably less bioactivity, with only two isolates (Tailings 4 and Tailings 7) exhibiting partial inhibition, and no isolate demonstrating strong inhibition against either pathogen. This discrepancy may reflect differences in heavy metal type, concentration or bioavailability between the two contaminated sites, potentially influencing microbial adaptation strategies.

Isolates from the organic rhizosphere, used as a control from uncontaminated soil, exhibited minimal inhibitory activity. Only two out of 13 isolates (Organic 5 and Organic 6) showed partial inhibition against a single pathogen. The overall lack of bioactivity among these samples reinforces the hypothesis that environmental stressors, such as heavy metal contamination, enhance the selective pressure required to induce antimicrobial secondary metabolite production.

These findings validate the use of metal‐contaminated rhizospheres as effective bioprospecting grounds for antimicrobial‐producing microorganisms. The results also justify the prioritisation of Steel 7 for UPLC‐MS analysis, as its dual‐pathogen inhibitory profile suggests a high potential for broad‐spectrum antimicrobial activity (Garbeva and de Boer 2009).

Multi‐drug‐resistant strains of *P. aeruginosa* have been tested in the literature using the Kirby–Bauer disc diffusion test with antibiotics tetracycline, cefixime, ciprofloxacin, erythromycin, meropenem, azithromycin, doxycycline, aztreonam, co‐trimoxazole and gentamycin (Mini et al. 2022). However, none of the tested antibiotics showed an inhibitory response alone. One of the only remaining ways to treat an infection by the same strain of *P. aeruginosa* is with a combination of ciprofloxacin and streptomycin, with cefotaxime (Abbas et al. 2020).

Likewise, *A. baumannii* is resistant to ceftazidime, gentamicin, ticarcillin, piperacillin, aztreonam, cefepime, ciprofloxacin, imipenem and meropenem, and only sensitive to amikacin and tobramycin (Mirzaei et al. 2020). This not only confirms the severity of the antibiotic resistance of the XDR pathogens tested but also highlights the potential of positive isolates in this study to secrete metabolites with broad‐spectrum antimicrobial activity. The best performing strains were isolated from steel wastes, designated as Steel 3, Steel 4 and Steel 7, and were investigated further.

### 3.4 Secondary Screening—Agar Well Diffusion Test

The agar diffusion assay was used as a secondary screening method to evaluate the antimicrobial activity of secondary metabolites extracted from the top‐performing isolates (Steel 3, Steel 4 and Steel 7) against *P. aeruginosa* and *A. baumannii* . The results are summarised in Table 4. Inhibition was observed only for extracts dissolved in DMSO and acetic acid, with no inhibition detected for extracts dissolved in acetone or ethyl acetate. As these agar diffusion assays were performed only once without biological replication, the data are presented qualitatively to indicate preliminary trends rather than statistically validated results. The promising antimicrobial profiles of Steel 3, Steel 4 and Steel 7 will require future replication and more rigorous statistical analysis to fully confirm their bioactive potential.

**TABLE 4:** Agar diffusion assay results showing preliminary inhibition zones (in mm) for secondary metabolites extracted from Steel 3, Steel 4 and Steel 7 against *P. aeruginosa* and *A. baumannii* ^a^.

Tested secondary metabolites	Inhibition against *P. aeruginosa* (mm)				Inhibition against *A. baumannii* (mm)			
	DMSO	Acetone	Ethyl acetate	Acetic acid	DMSO	Acetone	Ethyl acetate	Acetic acid
Steel 3	53	None	None	67	52	None	None	59
Steel 4	50	None	None	66	55	None	None	61
Steel 7	54	None	None	64	56	None	None	62
Control (pure solvent)	None	None	None	Inhibition	None	None	None	Inhibition

^a^ *n* = 1.

Steel 7 demonstrated the highest inhibition zones for both pathogens (54 mm against *P. aeruginosa* and 56 mm against *A. baumannii* in DMSO), reinforcing its identification as the most potent isolate in primary screening. Steel 3 and Steel 4 also exhibited strong inhibition, although slightly lower than Steel 7. Notably, inhibition observed with acetic acid extracts must be interpreted with caution, as the solvent control (pure acetic acid) alone demonstrated inhibitory effects, indicating that antimicrobial activity observed in this solvent may not be solely attributable to the bacterial metabolites. The acetic acid control was, therefore, excluded from assessment of extracts' inhibitory activity (Table 4). Considering the control and experimental results together, it was observed that DMSO did not exhibit any biological activity and can therefore be deemed appropriate to use in further investigations, as well as validating that the observed activity from the Agar Well diffusion test where DMSO was used as a solvent is as a result of activity of the dissolved secondary metabolites.

DMSO is well known for its capabilities as an excellent solvent of both polar and non‐polar natural compounds, and is often used within the drug discovery process as it has been shown to solubilise low‐solubility bioactive natural compounds (Nakhle et al. 2020), and has a relatively low level of toxicity compared to other organic solvents, as demonstrated in the above experiments and confirmed in the literature (Karim et al. 2023).

16S rRNA sequencing identified the three best‐performing isolates (Steel 3, Steel 4 and Steel 7) as belonging to the *Bacillus* genus, with sequence identities of 100%. Steel 3 and Steel 7 matched *Bacillus siamensis* / *B. velezensis* , while Steel 4 matched *Bacillus amyloliquefaciens* / *B. subtilis* as shown in Table 5 below. These results are consistent with the known ability of Bacillus species to produce a wide range of bioactive secondary metabolites, supporting their selection for further chemical characterisation (Nakayasu et al. 2023).

**TABLE 5:** Identification of the top‐performing bacterial isolates based on 16S rRNA sequencing.

Isolate name	Identification result	% sequence identity
Steel 3	*Bacillus siamensis / B. velezensis*	100
Steel 4	*Bacillus amyloliquefaciens / B. subtilis*	100
Steel 7	*Bacillus siamensis / B. velezensis*	100

*Note:* Results indicate the closest known species matches, with 100% sequence identity for all isolates. Taxonomic assignment at the species level should be interpreted cautiously given the inherent limitations of 16S rRNA resolution among closely related Bacillus species.

This analysis was done to compare any potential active compounds identified at later stages to natural product libraries to see if the specified species are known to secrete the identified compounds (Nakayasu et al. 2023). These findings further support the hypothesis that environmental stressors, particularly heavy metal contamination, select for microbial strains with enhanced secondary metabolite production, offering an enriched source for the discovery of novel antimicrobial agents.

### 3.5 UPLC‐HRMS Sample Preparation and Analysis

UPLC‐HRMS analysis was done to tentatively identify the active compounds within the crude extract, assuming that the active compounds would be in the highest concentrations. This is commonly performed on natural product extracts and bacterial secondary metabolite extracts, as seen in the literature, as a first step in the characterisation process (Maqbool et al. 2019; Thakur et al. 2024; Mohammed et al. 2021; Alkhatib et al. 2022). The fermentation of 1 L of selective enrichment medium produced a dry weight of 1.20 g of Steel 3 crude extract, 0.96 g of Steel 4 crude extract and 2.34 g of Steel 7 crude extract.

Solubility tests were run to ensure the best solvent for the samples was used. The detailed results are included in the Supporting Information, Table S2. The MeOH: H_2_O solvent in a ratio of 3:2 was able to dissolve all the crude extracts well and was selected as the best solvent for the UPLC‐HRMS analysis.

**FIGURE 2:**
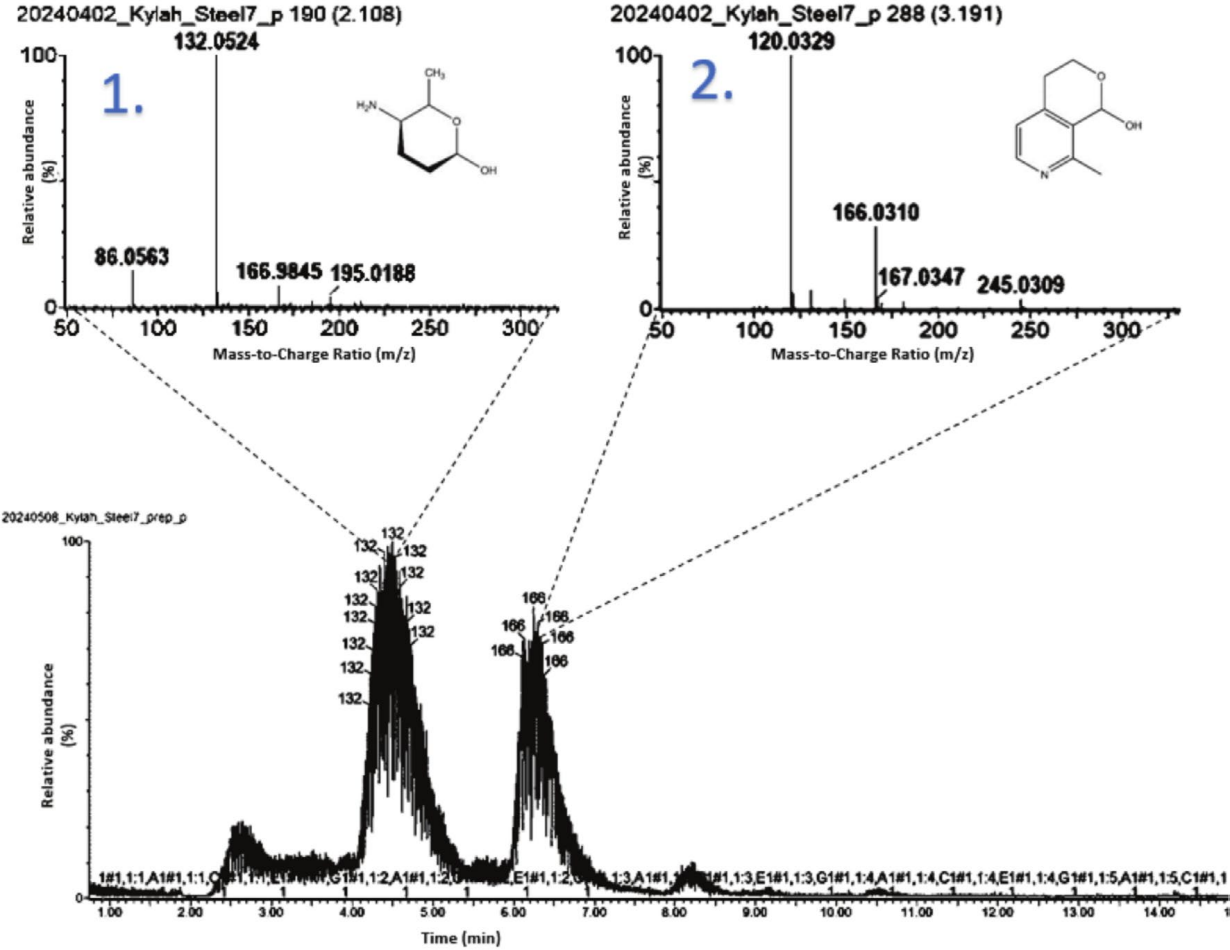
ESI positive mode chromatogram of filtered Steel 7 extract dissolved in MeOH:H_2_O (3:2) with their mass spectra attached. The tentatively identified structures show possible structures of the mass spectra represented.

UPLC‐HRMS was run on the Steel 3, Steel 4 and Steel 7 extracts, where Steel 7 showed the most activity in preliminary screening. Chromatograms and UV activities are provided for Steel 3, Steel 7 and Steel 4 (and the solvent blank) are provided in the Supporting Information as Figures S6–S12. The most prominent peaks of Steel 7's chromatogram are shown below in Figure 2, and their respective mass spectra are attached.

After investigation of the largest peaks visible using the methods stated above in section 2.8, the following compounds were tentatively identified as shown in Table 6.

**TABLE 6:** UPLC‐HRMS results of the most prominent peaks within the crude extracts of the microbial secondary metabolites provided.

Peak annotation	Monoisotopic molecular mass	Corrected molecular formula (MassLynx)	Adducts	Molecular mass error (mDa)	UV absorbance	Fragments	Tentative structure	Potential compound names	References
1.	132.1025	C_6_H_13_NO_2_	[M + H]^+^	−2.0	253.2961	86 45	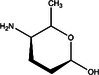	Tolyposamine (possible fragment of Tolypomycin)	(Kishi et al. 1972)
2.	166.0856	C_9_H_11_NO_2_	[M + H]^+^	−2.1	257.2960	120 46	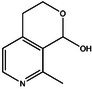	Gentiatibetine (possible fragment of Gentiopicrin)	(Šavikin et al. 2009)

The peak m/z 132.1025, annotated as (peak 1), is of great interest, as it was present within the global analysis. After investigation, as stated in the methodology section, a potential molecule with the name Tolypolyamine was identified. Tolypolyamine, a key component of Tolypomycin Y, an antibiotic discovered in 1972 (Kishi et al. 1972). Tolypomycin has previously been reported as a natural product isolated from *Streptomyces tolypophorus* species. However, all antimicrobial‐producing isolates in this work were identified as Bacillus species. While Streptomyces is a historically prolific source of bioactive secondary metabolites, it is plausible that Bacillus species may produce chemically similar or identical compounds through independent biosynthetic evolution. Such phenomena have been observed across various bacterial taxa, emphasising the untapped biosynthetic potential of Bacillus strains, particularly under extreme environmental stressors such as heavy metal contamination. Importantly, the historical association of this compound with microbial natural product biosynthesis primarily serves to confirm that the compound is biologically synthesizable, supporting its microbial origin in this study. Recent studies have highlighted the extensive biosynthetic capabilities of Bacillus species, particularly within the *B. subtilis* group, which are known to produce a diverse array of secondary metabolites, including non‐ribosomal peptides and polyketides (Iqbal et al. 2023). This growing recognition of Bacillus' metabolic versatility reinforces that the bioactive compounds identified in this study may originate from the Bacillus isolates. Tolypomycin was tested against multiple pathogens, notably a strain of *P. aeruginosa* IFO3080 (a much earlier and less resistant strain than strain ATCC 27853 used in this project) and showed inhibition (Kishi et al. 1972). Its mechanism includes disrupting bacterial cell integrity, exhibiting efficacy comparable to other broad‐spectrum antibiotics such as tetracycline and chloramphenicol (Kishi et al. 1972).

The peak showing a m/z 166.0856 (peak 2) was tentatively identified as Gentiatibetine. Gentiatibetine, extracted from *Gentiana lutea* in the literature, shows notable antimicrobial activity, particularly against Gram‐positive bacteria like *S. aureus* and *Streptococcus faecalis* , as well as some Gram‐negative strains, including *E. coli* and *P. aeruginosa* . Studies reveal that Gentiatibetine's effectiveness is reflected in low minimum inhibitory concentration (MIC) values, suggesting it could serve as a potent antimicrobial agent. Its activity against a broad spectrum of pathogens supports its therapeutic potential, potentially overcoming certain resistant bacterial strains. It is a part of the antibiotic Gentiopicrin that is found in medicinal plants in China (Šavikin et al. 2009). Studies have shown that Gentiopicrin can inhibit the growth of the exact same strain of *P. aeruginosa* (ATCC 27853) that this study tested against, thereby contributing towards confirming the tentative identification of the active compounds within the rhizospheric secondary metabolites crude extract (Šavikin et al. 2009).

## 4 Conclusion and Recommendations

This study explored the rhizospheric bacteria associated with eight plants growing under heavy‐metal stress in a contaminated scrapyard environment in Pretoria, South Africa. The results demonstrated that these microorganisms secrete secondary metabolites with notable antimicrobial activity against *P. aeruginosa* ATCC 27853 and *A. baumannii* ATCC‐BAA‐1605, two critically ranked antibiotic‐resistant pathogens. The active compounds tentatively identified include Tolyposamine and Gentiatibetine. Further validating these metabolites' novelty and potential biomedical applications requires advanced techniques, such as UPLC‐MS and Nuclear Magnetic Resonance (NMR), and determining their half‐maximal inhibitory concentration (IC_50_). These steps are essential to warrant further investigations into their pivotal contributions to biomedicine and biopharmaceutics. It is also recommended that once a full characterisation is completed, the synthesis or bulk production of these antibiotic compounds should be explored. The scalability and synthetic feasibility of the identified metabolites remain subjects for future research.

Several limitations must be acknowledged when interpreting these findings. The study was limited to a small number of environmental sampling sites, restricted to two contaminated locations and one set of organic soil. Additionally, rhizospheric samples from each site were pooled, focusing on the effect of environmental stress rather than plant‐specific microbial interactions. Solubility testing and antimicrobial assays were exploratory and qualitative, conducted without biological replication or quantitative solubility measurements. Furthermore, mass spectrometry‐based metabolite identification was preliminary, pending full structural confirmation.

Despite these constraints, the study demonstrated that extreme environmental stress, specifically heavy‐metal contamination, can induce rhizospheric microorganisms to secrete bioactive antimicrobial compounds. As a first conceptual exploration into this strategy, the findings establish a valuable foundation for future work involving expanded sampling across diverse contaminated environments, rigorous quantitative analyses and full structural characterisation of active metabolites.

Future research should also investigate how heavy‐metal‐induced stress might influence the resistance mechanisms of clinically relevant pathogens, including the potential cross‐resistance between heavy‐metal tolerance and antibiotic resistance. Such studies could offer deeper insights into the interplay between environmental stressors and the evolution of antimicrobial resistance.

In conclusion, this study highlights the untapped potential of heavy‐metal‐contaminated rhizospheres as a promising source of novel antimicrobial agents and underscores the importance of further exploration into environmentally driven strategies for combating antibiotic resistance.

## Author Contributions

**Kylah B. Millard:** investigation, analysis of results, validation, visualisation, writing – original draft. **John O. Unuofin:** conceptualisation, supervision, writing – review and editing. **Samuel A. Iwarere:** financial support, supervision, resources, writing – review and editing. **Luke Invernizzi:** supervision, data curation, writing – review and editing. **Michael O. Daramola:** supervision, writing – review and editing.

## Conflicts of Interest

The authors declare no conflicts of interest.

## Supporting information


**Data S1:** Supporting Information.

## Data Availability

The data that supports the findings of this study is available in the Supporting Information of this article.
