# Supplementary material for: Bioprospecting Heavy‐Metal Rhizospheres for Novel Therapeutics Against High‐Priority Multi‐Drug‐Resistant Pseudomonas aeruginosa and Acinetobacter baumannii : A Case of Toxic to Treatment
Source: Environ Microbiol Rep. 2025 Sep 18;17(5):e70182. doi: 10.1111/1758-2229.70182 (PMC12444940; doi:10.1111/1758-2229.70182)
Supplement: Supplementary file 1 — Data S1: Supporting Information. [file EMI4-17-e70182-s001.docx]

**Supplementary material**

**Bioprospecting Heavy Metal Rhizospheres for Novel Therapeutics Against High-Priority Multi-Drug-Resistant *Pseudomonas aeruginosa* and *Acinetobacter baumannii*:** **A Case of Toxic to Treatment**

Kylah B. Millard­­^1^, John O. Unuofin^1^, Luke Invernizzi^2^, Michael O. Daramola^1^, Samuel A. Iwarere*^1^

^1^Sustainable Energy and Environment Research Group (SEERG), Department of Chemical Engineering, Faculty of Engineering, Built Environment and Information Technology (EBIT), University of Pretoria, Hatfield, Pretoria 0002, South Africa.

^2^Biodiscovery Centre, Department of Chemistry, Faculty of Natural and Agricultural Sciences (NAS), University of Pretoria, Hatfield, Pretoria 0002, South Africa.

*Corresponding author: [samuel.iwarere@up.ac.za](mailto:samuel.iwarere@up.ac.za); Tel.: +27 (0)12 420 3092

**Table S1:** X-ray Fluorescence (XRF) to determine the elemental composition of the soil from the Steel, Tailings and organic harvest sites

|  | **Description** | **Steel dump site soil** | **Tailings dump site soil** | **Organic soil** |
| --- | --- | --- | --- | --- |
| **Ag** | ppm | 1,5 | 1.7 | 1.5 |
| **Al** | % | 5.863 | 6.52 | 4.166 |
| **As** | ppm | 24.3 | 9.2 | 3.4 |
| **Ba** | ppm | 188.1 | 352.1 | 152.1 |
| **Bi** | ppm | 0.9 | <0.3 | 0.4 |
| **Br** | ppm | 8.4 | 4 | 5.7 |
| **Ca** | % | 0.8744 | 3.536 | 0.9855 |
| **Cd** | ppm | 1 | 1.6 | <0.1 |
| **Ce** | ppm | 20.8 | 27.1 | <1.5 |
| **Cl** | ppm | 201.7 | 141.3 | 124.6 |
| **Co** | ppm | <1.0 | 96 | <1.0 |
| **Cr** | ppm | 643.8 | 657.5 | 166.9 |
| **Cs** | ppm | <0.8 | <0.8 | <0.8 |
| **Cu** | ppm | 152.1 | 207.8 | 46.6 |
| **Fe** | % | 4.204 | 19.8 | 3.718 |
| **Ga** | ppm | 9.5 | 10.2 | 8.1 |
| **Ge** | ppm | 1.2 | 1.9 | 0.6 |
| **Hf** | ppm | <0.9 | <7.5 | <2.8 |
| **Hg** | ppm | <0.1 | <0.1 | <0.1 |
| **I** | ppm | <0.6 | <0.6 | <0.6 |
| **In** | ppm | 1.4 | 1.1 | <0.3 |
| **K** | % | 0.3587 | 0.4237 | 0.7156 |
| **La** | ppm | 15.7 | 7.6 | <1.5 |
| **Mg** |  | 0.3872 | 2.282 | 0.1687 |
| **Mn** | ppm | 594.2 | 4181 | 567.5 |
| **Mo** | ppm | 4.2 | 5.5 | 1 |
| **Na** |  | <0.0020 | <0.0020 | <0.0020 |
| **Nb** | ppm | 9.7 | 7.6 | 6.3 |
| **Ni** | ppm | 73.9 | 167.2 | 75.3 |
| **P** | % | 0.08113 | 0.04658 | 0.04424 |
| **Pb** | ppm | 103.8 | 76.3 | 18.5 |
| **Rb** | ppm | 29.9 | 19.8 | 43 |
| **S** | ppm | 1101 | 881.4 | 438.1 |
| **Sb** | ppm | 4.8 | <1.2 | <0.4 |
| **Se** | ppm | 0.3 | 0.6 | 0.2 |
| **Si** | % | 18.05 | 17.98 | 13.82 |
| **Sn** | ppm | 5.8 | 9.3 | <0.7 |
| **Sr** | ppm | 89.3 | 71.6 | 42.2 |
| **Ta** | ppm | <0.8 | <0.8 | <0.8 |
| **Te** | ppm | <0.5 | <0.5 | <0.5 |
| **Th** | ppm | 7.3 | 5.1 | 5.4 |
| **Ti** | % | 0.3715 | 0.3926 | 0.1507 |
| **Tl** | ppm | <0.2 | <0.2 | <0.2 |
| **U** | ppm | 2.1 | 1.9 | 1.9 |
| **V** | ppm | 100 | 282.5 | 62.5 |
| **W** | ppm | 3.1 | 7.6 | 2.4 |
| **Y** | ppm | 16.2 | 19.4 | 10.4 |
| **Zn** | ppm | 262.7 | 501 | 111.6 |
| **Zr** | ppm | 160.5 | 153.2 | 96.6 |

**Table S2:** Solubility results of the crude extracts in different solvents and solvent ratios.

| Bacterial origin of crude extract | MeOH | H_2_O | ACN | MeOH:H_2_O  1:1 | MeOH:H_2_O  1:2 | MeOH:H_2_O  3:2 |
| --- | --- | --- | --- | --- | --- | --- |
| Steel 3 | Soluble | Soluble | Insoluble | Soluble | Very soluble | Very soluble |
| Steel 4 | Slightly soluble | Soluble | Insoluble | Soluble | Soluble | Very soluble |
| Steel 7 | Barely soluble | Very soluble | Insoluble | Very soluble | Soluble | Very soluble |

The solubility assessments were conducted qualitatively by visual inspection of dissolution, without quantitative mass determination.

**X-ray diffraction (XRD) analysis of the contaminated soil before and after synthetic contamination**


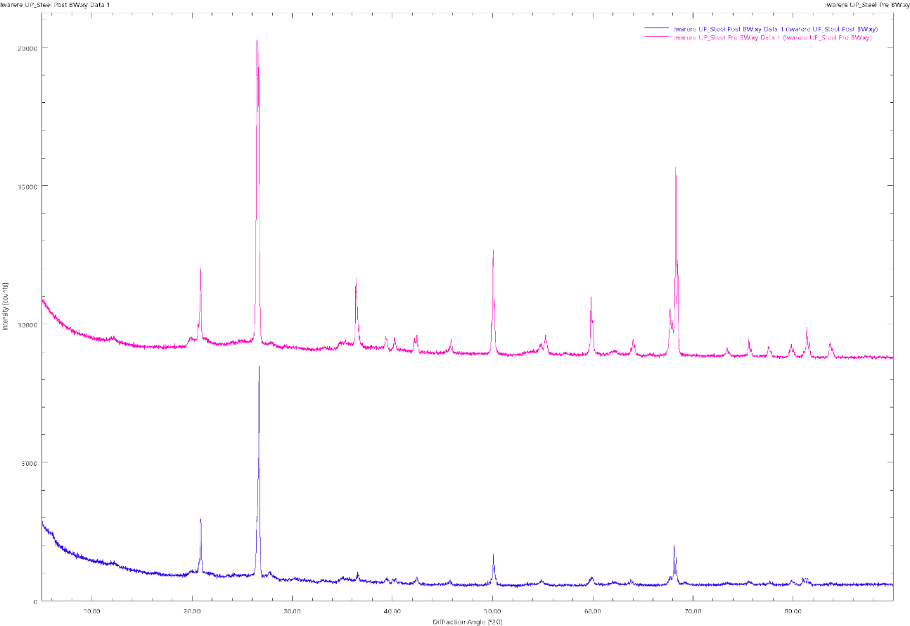


**Figure S1:** X-ray diffraction (XRD) patterns of rhizospheric soil samples before (pink) and after (blue) exposure to battery waste. The X-axis represents the diffraction angle (2θ, degrees), and the Y-axis represents the relative intensity (arbitrary units, a.u.).


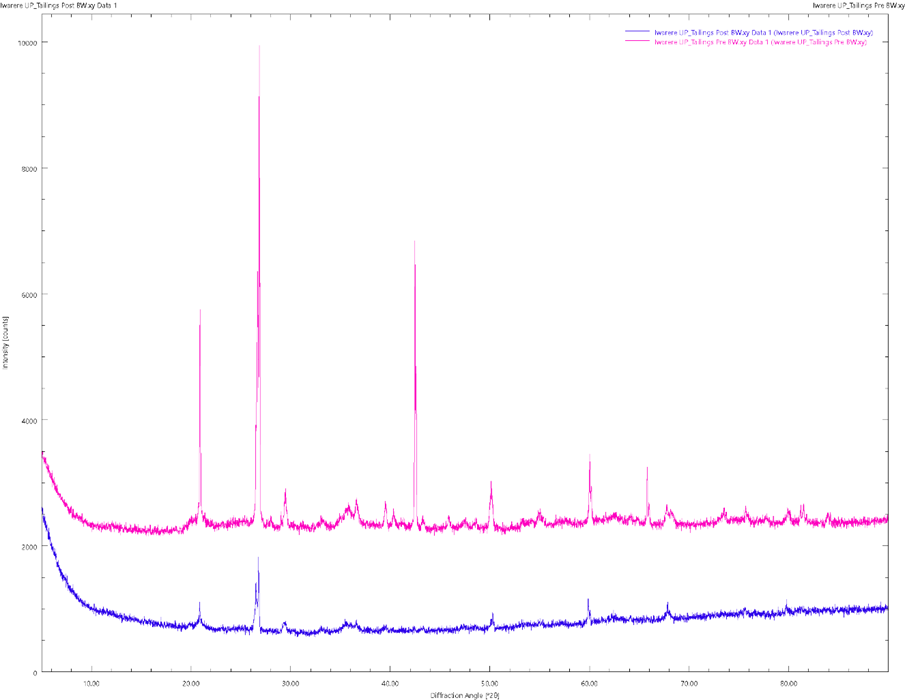


**Figure S2:** X-ray diffraction (XRD) patterns of rhizospheric soil samples before (pink) and after (blue) exposure to battery waste. The X-axis represents the diffraction angle (2θ, degrees), and the Y-axis represents the relative intensity (arbitrary units, a.u.).

**Plants used as experiments and as a control:**


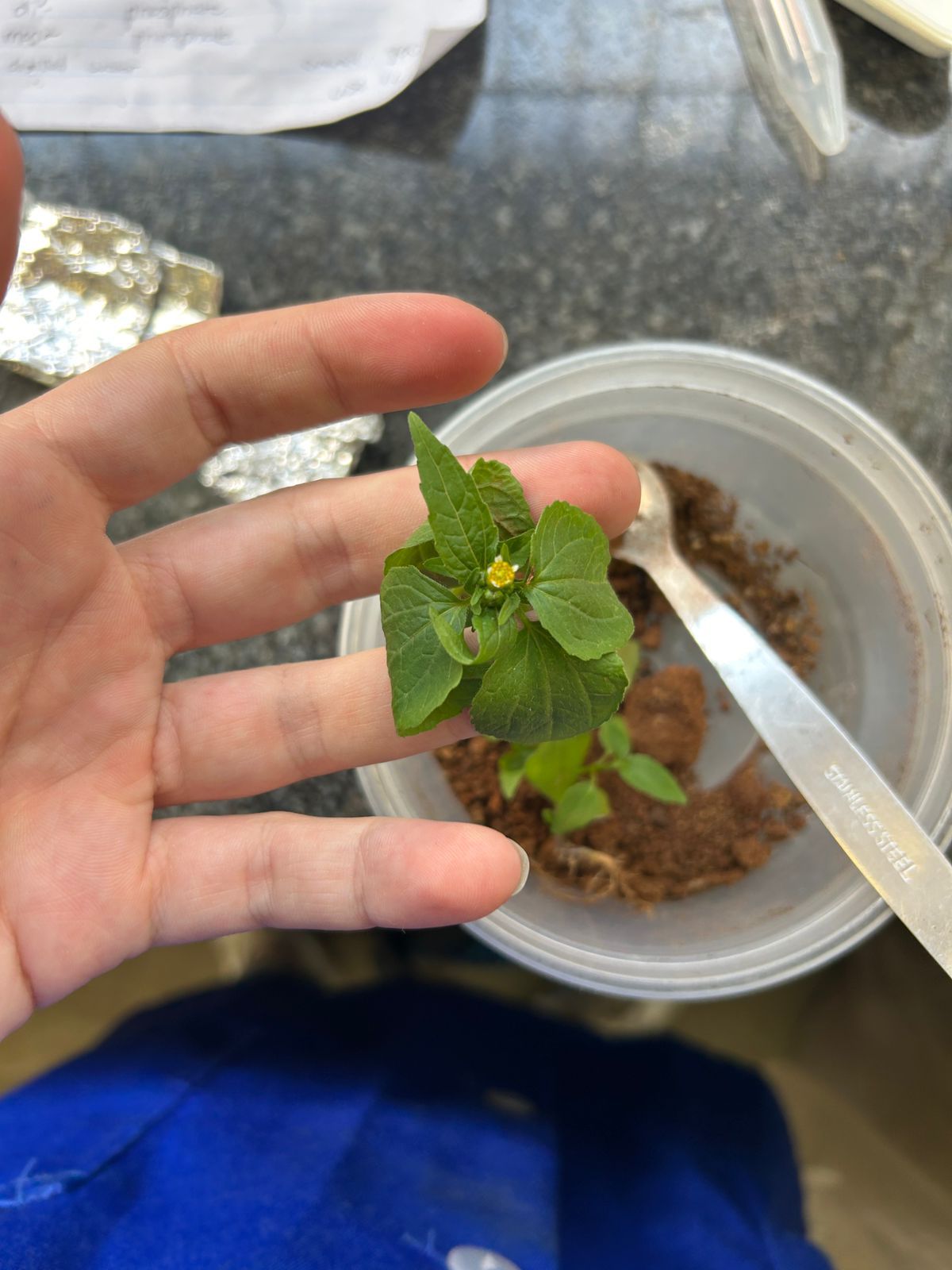


**Figure S3:** Lantana sp


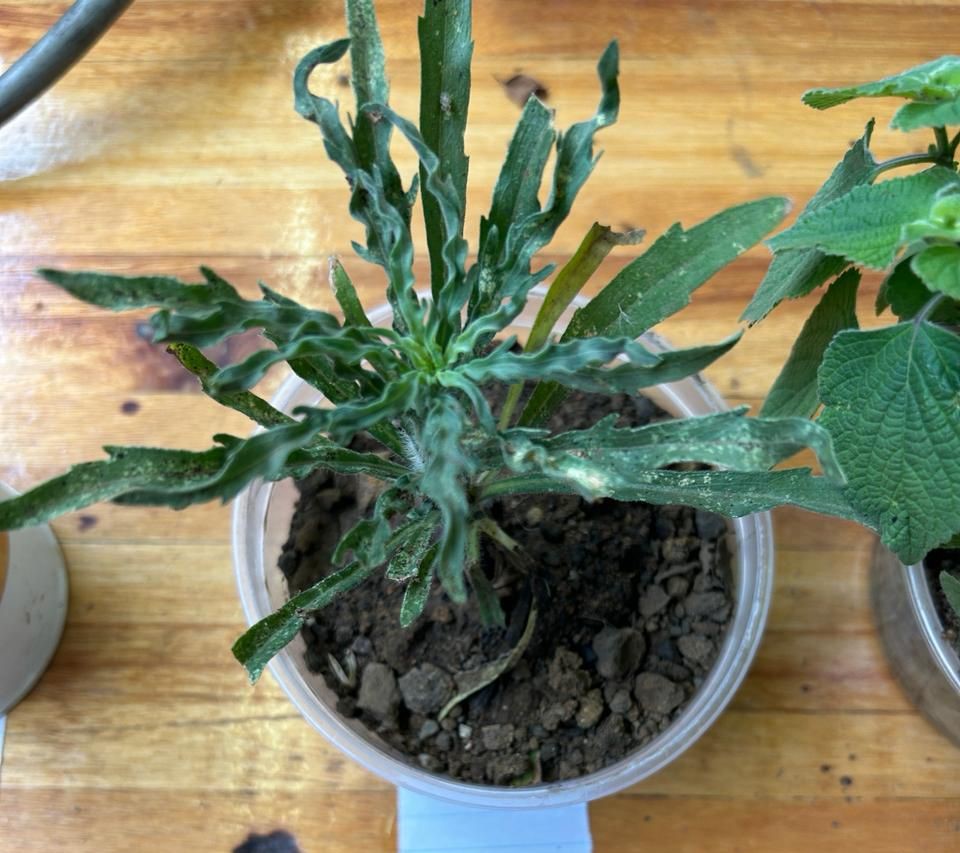

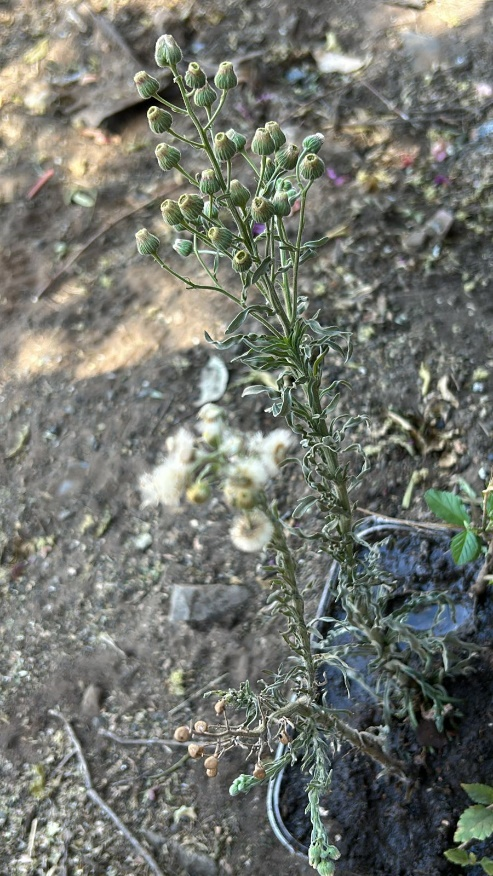


**Figure S4:** Erigeron bonariensis

**Figure S5:** Erigeron bonariensis


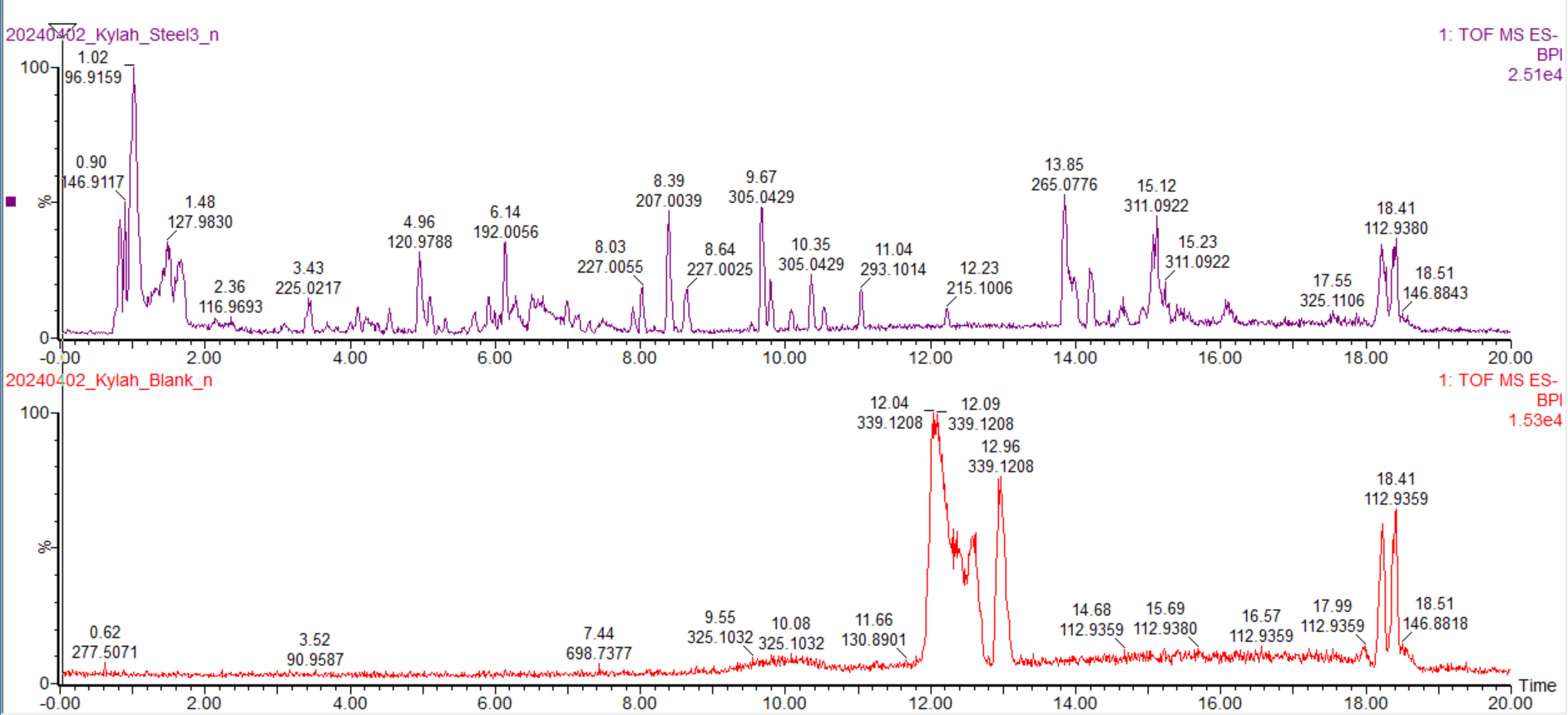


**Figure S6:** UPLC-HRMS chromatogram of Steel 3 extract and solvent blank acquired in ESI negative mode. The X-axis represents retention time (minutes), and the Y-axis represents relative intensity (arbitrary units, a.u.).


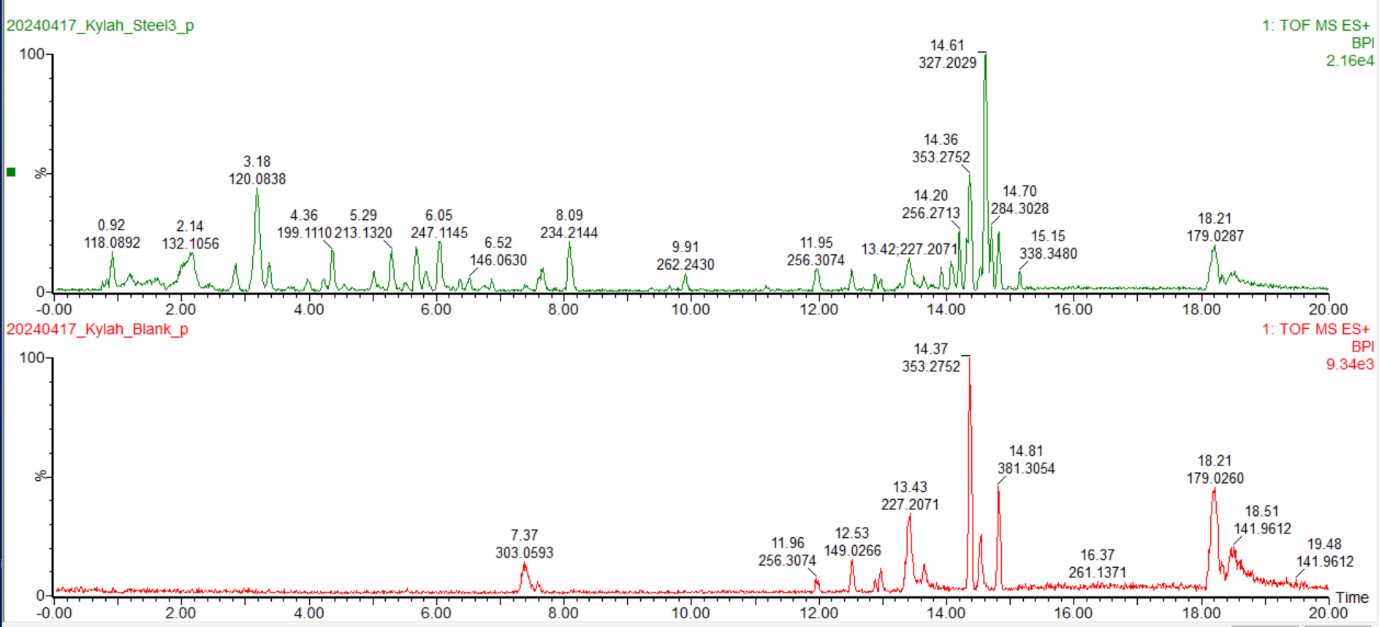


**Figure S7:** UPLC-HRMS chromatogram of Steel 3 and solvent blank in ESI positive mode. The X-axis represents retention time (minutes), and the Y-axis represents relative intensity (arbitrary units, a.u.).


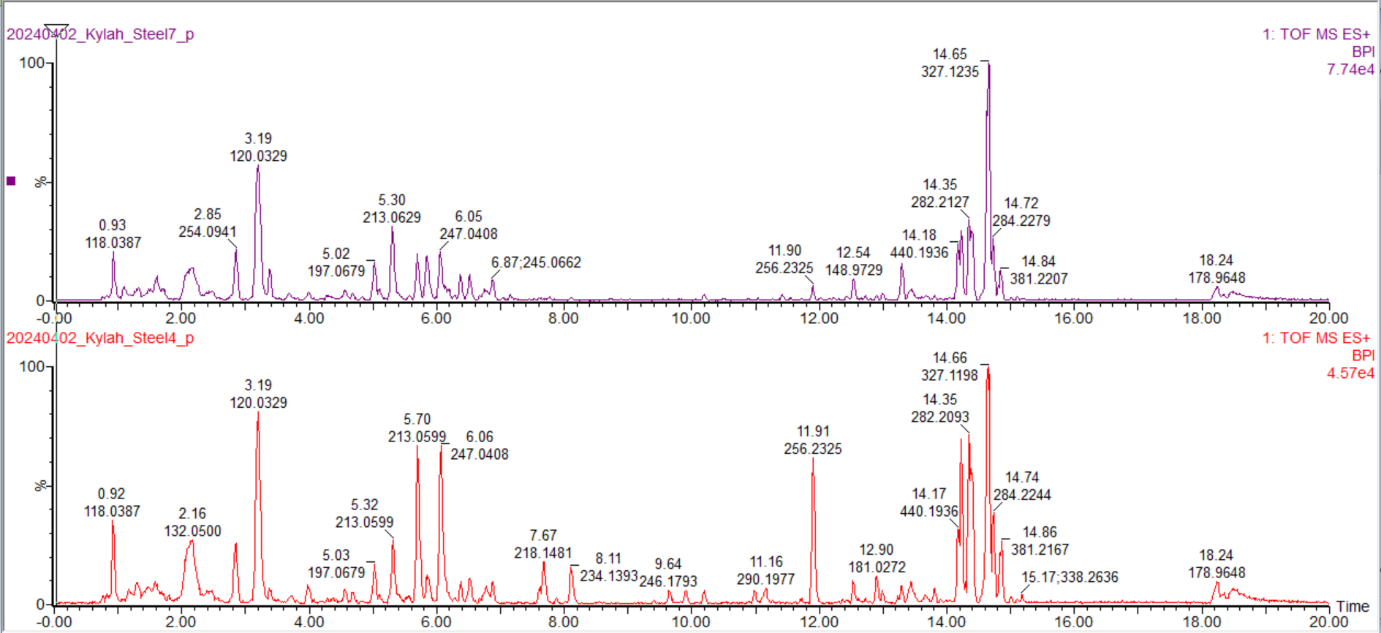


**Figure S8:** UPLC-HRMS chromatogram of Steel 7 and Steel 4 in ESI positive mode. The X-axis represents retention time (minutes), and the Y-axis represents relative intensity (arbitrary units, a.u.).


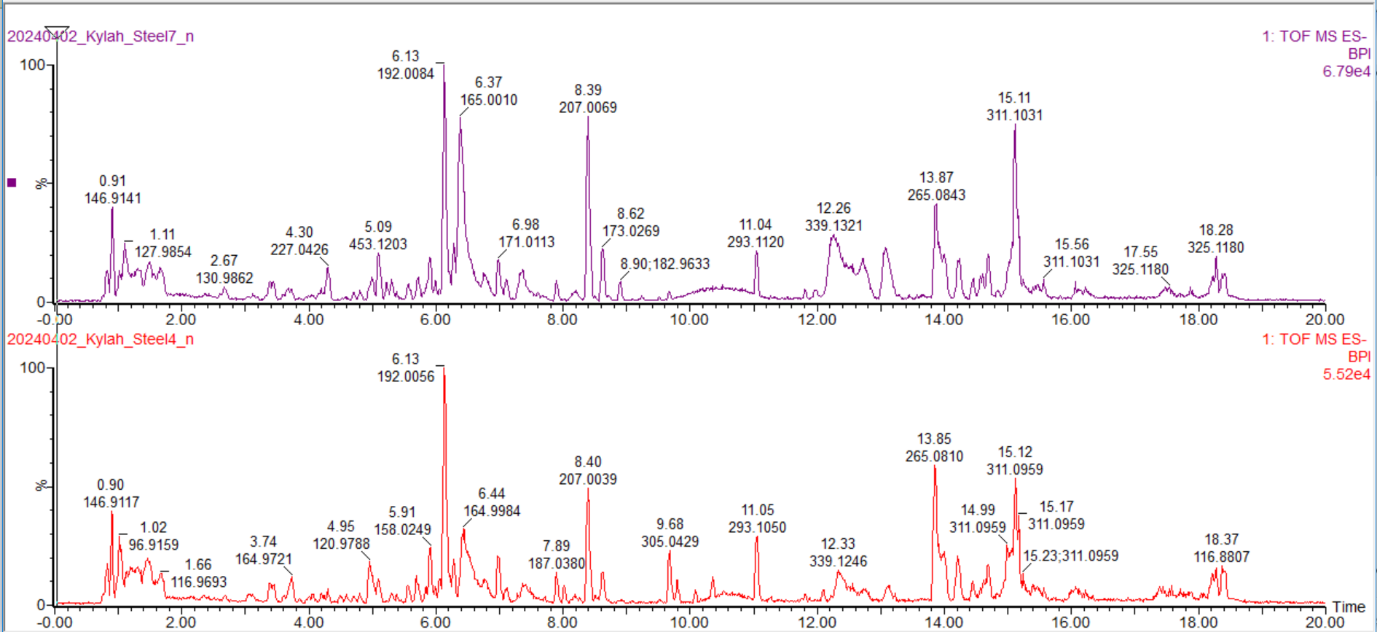


**Figure S9:** UPLC-HRMS chromatogram of Steel 7 and Steel 4 in ESI negative mode. The X-axis represents retention time (minutes), and the Y-axis represents relative intensity (arbitrary units, a.u.).


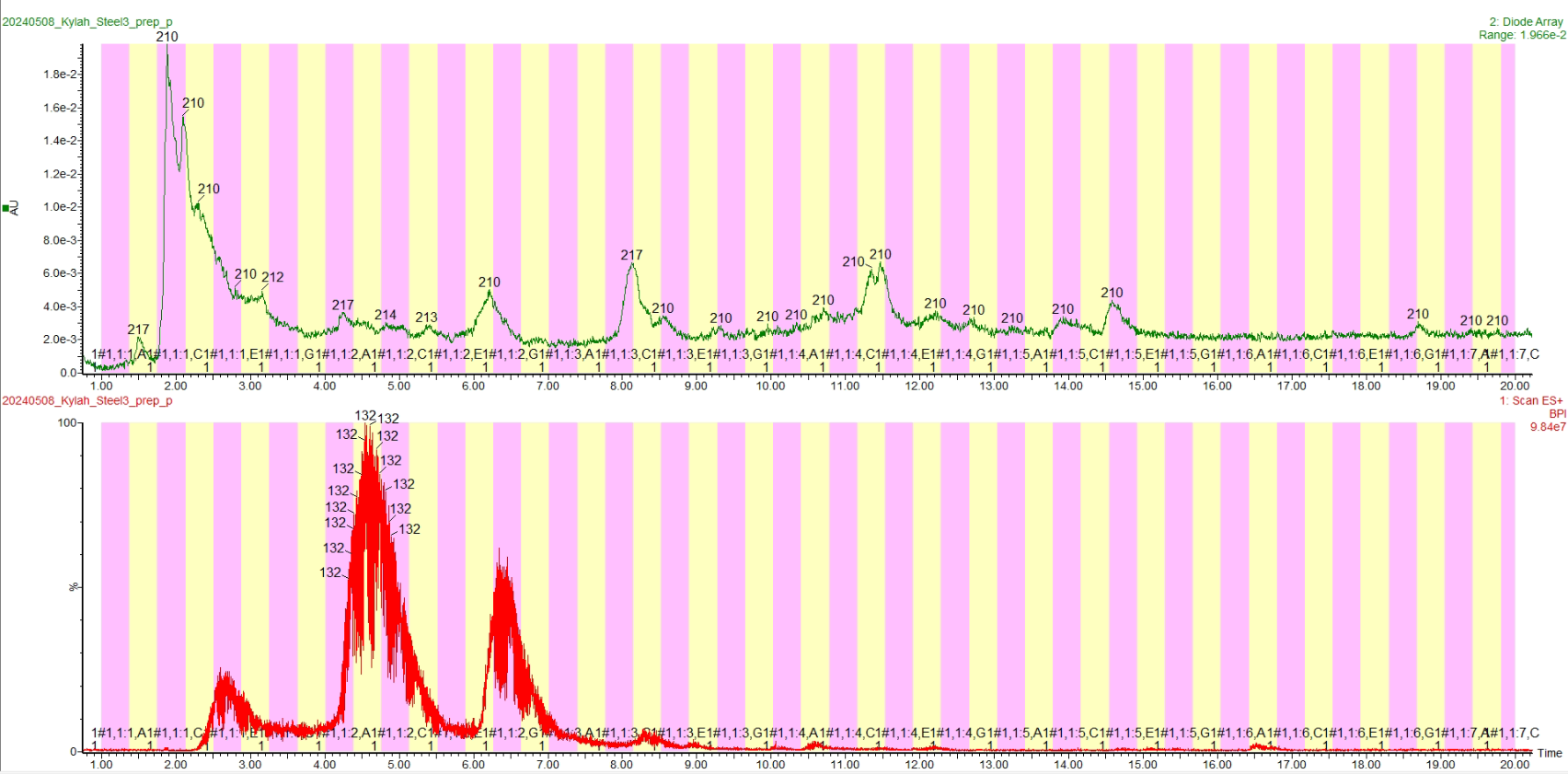


**Figure S10:** HPLC chromatogram and UV activity of Steel 3 in ESI positive mode. The X-axis represents retention time (minutes), and the Y-axis represents relative intensity (arbitrary units, a.u.).


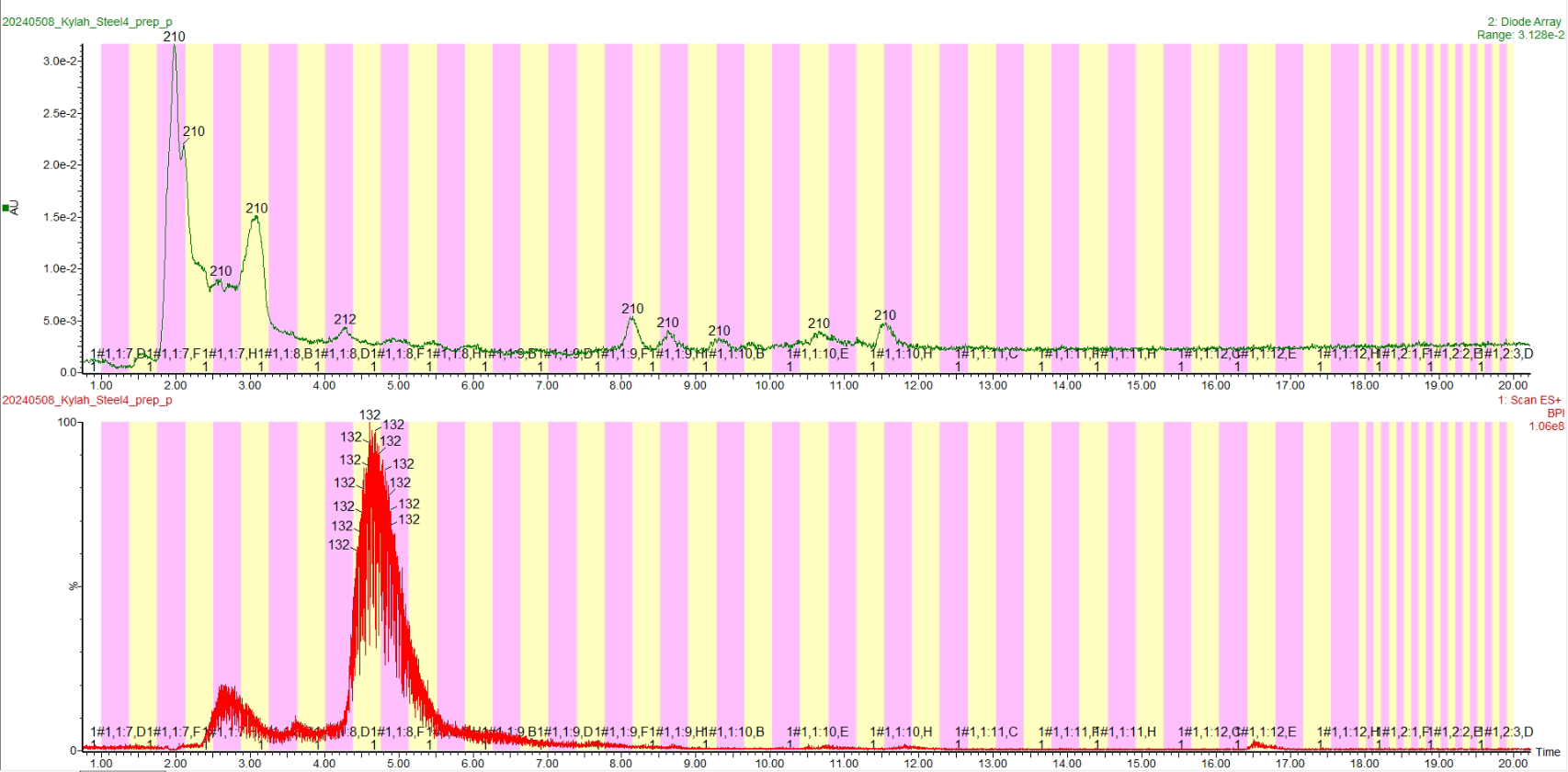


**Figure S11:** HPLC chromatogram and UV activity of Steel 4 in ESI positive mode. The X-axis represents retention time (minutes), and the Y-axis represents relative intensity (arbitrary units, a.u.).


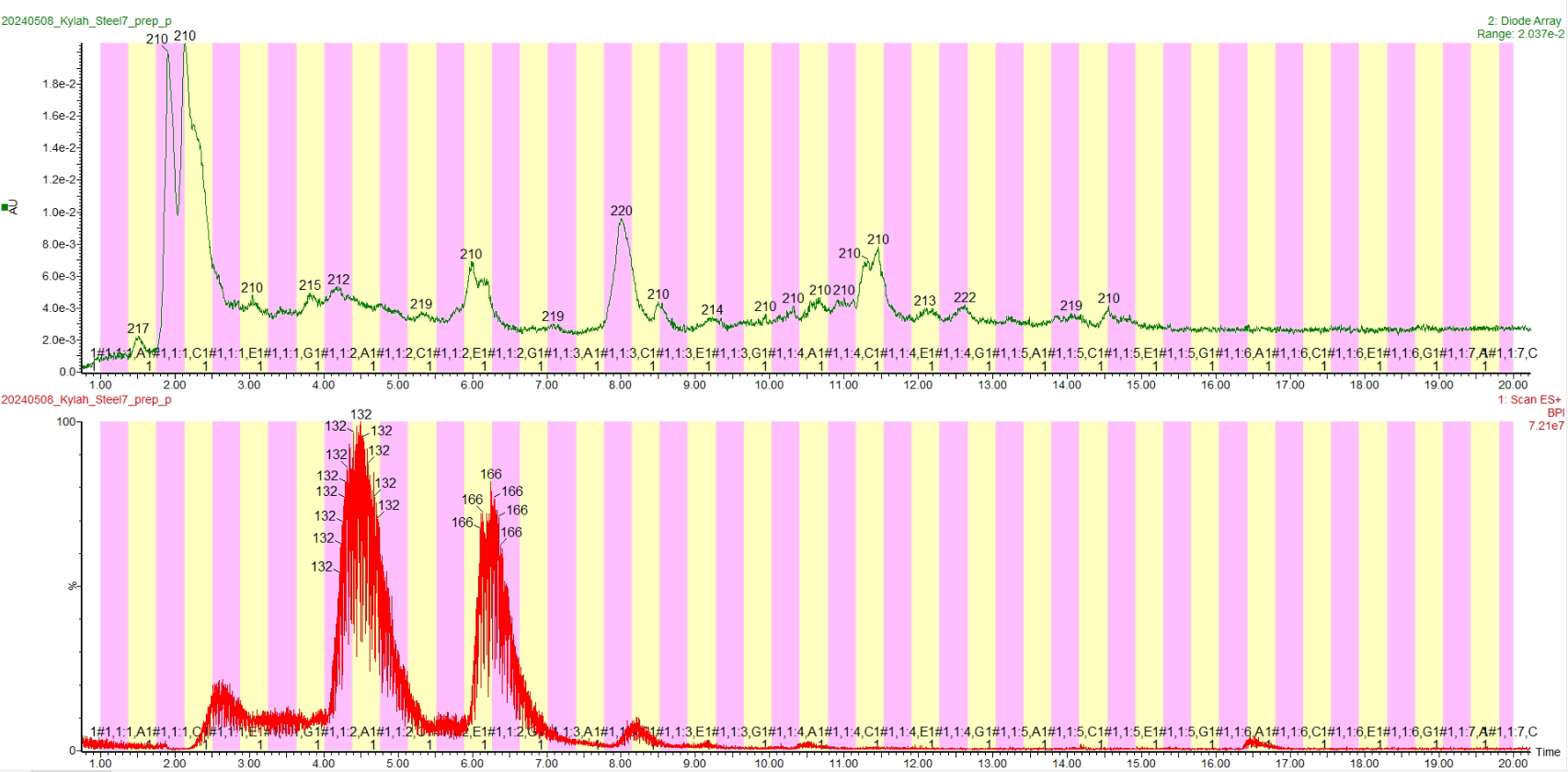


**Figure S12:** HPLC chromatogram and UV activity of Steel 7 in ESI positive mode. The X-axis represents retention time (minutes), and the Y-axis represents relative intensity (arbitrary units, a.u.).
